# Interest Among Primary Care Patients in Group Problem-Solving Gameplay for Mental Health

**DOI:** 10.5888/pcd15.170488

**Published:** 2018-06-21

**Authors:** Brandon J. Auer, Christopher Sciamanna, Joshua M. Smyth, Cristina I. Truica, Leah V. Cream, Dahlia Mukherjee

**Affiliations:** 1 Department of Medicine, Division of Population Health Research and Development, Pennsylvania State University College of Medicine, Hershey, Pennsylvania; 2 Department of Biobehavioral Health, Pennsylvania State University, University Park, Pennsylvania; 3 Division of Hematology-Oncology, Penn State Milton S. Hershey Medical Center and Penn State Cancer Institute, Hershey, Pennsylvania; 4 Department of Psychiatry, Penn State Milton S. Hershey Medical Center, Hershey, Pennsylvania

## Abstract

Mental health programs to improve problem-solving skills and reduce stress through social gameplay can improve psychiatric outcomes, but little is known about whether adult patients are interested in using them. Primary care patients (n = 467) completed a cross-sectional survey to assess interest in using 2 types of group programs for mental health. A significantly greater percentage (23.7%) of patients expressed interest in a gameplay-based program than in interpersonal therapy (17.6%) (*P* < .001). Lonely patients and younger patients were more likely to report interest in gameplay. Results suggest that diverse patient populations are interested in using gameplay programs for mental health.

## Objective

Although the prevalence of mental health disorders in primary care is estimated to be 20% to 55% internationally (1), patients — many of whom potentially have one or more distressing, comorbid chronic conditions (eg, diabetes, coronary artery disease) — frequently fail to receive treatment because of barriers to care associated with traditional interventions (eg, stigma, low enjoyment, cost) (2). Design thinking, a tool that encourages intervention designers to consider the emotional experience of the end user (3), can adapt effective interventions such as problem-solving therapy (4) to overcome such common barriers. Many commercially available games create problem-solving situations that can be integrated into an enjoyable, beneficial social experience for patients. We conducted this study to understand whether patients would report interest in such a novel game-based intervention.

## Methods

We obtained data from a quality improvement survey administered to outpatients at a general internal medicine outpatient clinic at the Penn State Hershey Medical Center in September 2016. Two questions were designed to assess program interest: “Would you consider participating in any of the following programs if offered for free? 1) A program where you and other people would play a game that can improve your problem-solving skills to help you feel less stressed? 2) A program where you and other people would discuss life problems with a mental health professional to help you feel less stressed?” Response options were yes or no. Demographic and health-related explanatory variables were assessed by using questions from the 2013 Centers for Disease Control and Prevention’s Behavioral Risk Factor Surveillance System (5). Mental health items were assessed by using the Patient Health Questionnaire-4 (PHQ-4) (6), and loneliness was assessed by using a modified version of the UCLA Three-Item Loneliness Scale (7). For PHQ-4 depression and anxiety subscales, scores of 3 or greater were considered positive for screening purposes (6). Loneliness items included the following: “How often do you feel that you lack companionship?,” “How often do you feel left out?,” and “How often do you feel isolated from others?” Response options were 1 (hardly ever), 2 (some of the time), and 3 (often). Loneliness was defined as answering any response but “hardly ever” for any loneliness question.

We used logistic regressions to describe associations between program interest and covariates. Adjusted odds ratios and 95% confidence intervals from logistic regression models were used to control for potential confounders. Analyses were performed in SPSS version 24.0 (IBM Corp). This study was exempt from review by the Penn State College of Medicine Institutional Review Board. We hypothesized, based on design thinking, that interest would be greater in a game-based intervention.

## Results

Of the 500 consecutive patients given a survey, 467 surveys were returned (response rate, 93.4%). Most participants were white, middle-aged, female, and college-educated (Table 1). The most common health-related comorbidities were overweight/obesity, high cholesterol, and hypertension. Overall, 33.1% of patients reported loneliness, 9.3% reported anxiety, and 5.3% reported depression. Of the patients answering the question about program interest, 11.8% (51 of 432) reported interest in only the gameplay program, 5.4% (23 of 429) reported interest in only the group interpersonal therapy program, 11.9% (51 of 430) reported interest in both programs, and 70.5% (304 of 431) reported interest in neither program. Overall interest in using a gameplay program (23.7% [102 of 430]) was higher than overall interest in a group interpersonal therapy program (17.6% [76 of 431]) (McNemar test, *P* < .001).

**Table 1:** Characteristics of a Sample of Primary Care Patients in Central Pennsylvania, 2016^a^

Variable	% (No.)		
	Overall	Interest in Gameplay Program^b^ Only	Interest in Group Interpersonal Therapy Program^c^ Only
**All**	100.0 (467)	11.8 (51)	5.4 (23)
**Age, y**			
18–40	10.0 (45)	5.9 (3)	17.4 (4)
41–50	11.6 (52)	9.8 (5)	21.7 (5)
51–60	18.5 (83)	25.5 (13)	17.4 (4)
61–70	26.5 (119)	41.2 (21)	30.4 (7)
>70	33.4 (150)	17.6 (9)	13.0 (3)
**Sex**			
Male	30.8 (139)	33.3 (17)	18.2 (4)
Female	69.2 (313)	66.7 (34)	81.8 (18)
**Race**			
White	95.1 (424)	92.2 (47)	91.3 (21)
Nonwhite	4.9 (22)	7.8 (4)	8.7 (2)
**Ethnicity**			
Hispanic	2.2 (10)	2.0 (1)	4.5 (1)
Non-Hispanic	97.8 (438)	98.0 (50)	95.5 (21)
**Education**			
No college	27.8 (125)	22.4 (11)	22.7 (5)
Some college (1–3 y)	24.7 (111)	20.4 (10)	18.2 (4)
College graduate (≥4 y)	47.4 (213)	57.1 (28)	59.1 (13)
**Smoking status**			
Smoker	4.7 (21)	2.1 (1)	0
Nonsmoker	95.3 (422)	97.9 (47)	100.0 (22)
**Chronic conditions**			
Have hypertension	56.7 (258)	71.4 (35)	47.8 (11)
Have diabetes	22.0 (100)	26.0 (13)	21.7 (5)
Have high cholesterol	53.9 (244)	54.0 (27)	39.1 (9)
History of heart attack or coronary artery disease	12.7 (58)	12.0 (6)	13.0 (3)
**Body mass index^d^**			
>30.0	36.0 (150)	48.9 (23)	50.0 (10)
25.0–30.0	35.0 (146)	29.8 (14)	20.0 (4)
18.5–24.9	29.0 (121)	21.3 (10)	30.0 (6)
**Self-reported health**			
Excellent, very good, or good	78.8 (361)	82.0 (41)	69.6 (16)
Fair or poor	21.2 (97)	18.0 (9)	30.4 (7)
**Functional ability**			
Ability to go up and down stairs at a normal pace	75.5 (348)	80.4 (41)	73.9 (17)
Ability to do chores (eg, vacuuming, yard work)	84.1 (387)	82.4 (42)	91.3 (21)
Ability to walk for ≥15 min	79.3 (365)	84.3 (43)	87.0 (20)
**Loneliness^e^**	33.1 (144)	40.0 (20)	45.5 (10)
Lack companionship	25.8 (113)	34.0 (17)	27.3 (6)
Feel left out	20.4 (89)	28.0 (14)	27.2 (6)
Feel isolated from others	19.9 (87)	30.0 (15)	18.2 (4)
**Depression^f^**	5.3 (24)	0	14.3 (3)
Feel down, depressed, or hopeless	24.4 (111)	21.6 (11)	50.0 (11)
Little interest or pleasure in doing things	17.6 (80)	11.8 (6)	40.8 (9)
**Anxiety^f^**	9.3 (42)	5.9 (3)	26.1 (6)
Feeling nervous, anxious, or on edge	33.2 (151)	29.5 (15)	56.5 (13)
Not being able to stop worrying	26.1 (122)	21.6 (11)	43.5 (10)
**Mental health symptom severity^f^**			
Normal	80.3 (359)	82.4 (42)	66.7 (14)
Mild	13.4 (60)	15.7 (8)	14.3 (3)
Moderate	3.8 (17)	2.0 (1)	9.5 (2)
Severe	2.5 (11)	0	9.5 (2)
**Group program interest**			
Interested in a gameplay program to reduce stress and enhance problem solving	23.7 (102)	100.0 (51)	0
Interested in a group talk therapy program with a mental health professional	17.6 (76)	0	100.0 (23)

a Data obtained from a quality improvement survey administered to outpatients at a general internal medicine outpatient clinic at the Penn State Hershey Medical Center in September 2016. Percentages are based on the number of survey participants who answered question; thus, denominators for percentages varied.

b Described in survey as “A program where you and other people would play a game that can improve your problem-solving skills to help you feel less stressed.”

c Described in survey as “A program where you and other people would discuss life problems with a mental health professional to help you feel less stressed.”

d Body mass index calculated as weight in kilograms divided by height in meters squared (kg/m^2^); weight and height were self-reported.

e Assessed by using a modified version of the UCLA Three-Item Loneliness Scale (7).

f Assessed by using the Patient Health Questionnaire-4 (6).

In multivariable logistic regression models of gameplay program interest, adjusted for all explanatory variables, younger patients (51–70 y vs >70 y), lonely patients (vs not lonely), and patients with a history of heart attack or coronary artery disease (vs no history) had significantly greater interest in the gameplay program (Table 2). In multivariable logistic regression models of group interpersonal therapy, adjusted for all explanatory variables, younger patients (51–60 y vs >70 y) and lonely patients (vs not lonely) were significantly more interested in the group interpersonal therapy program. No other explanatory variables were significant predictors of program interest. Eight of 21 (38.1%) patients with depression and 13 of 40 (32.5%) patients with anxiety were interested in the gameplay program. We found no evidence of multicollinearity (variance inflation factor <3 for all explanatory variables).

**Table 2:** Association Between Patient Characteristics and Interest in 2 Types of Mental Health Programs, Sample of Primary Care Patients in Central Pennsylvania (n = 467), 2016^a^

Characteristics	Gameplay Program^b^			Group Interpersonal Therapy Program^c^		
	Interested in Using, %^d^ (No.)	Unadjusted OR (95% CI)	Adjusted OR (95% CI)^e^	Interested in Using, %^d^ (No.)	Unadjusted OR (95% CI)	Adjusted OR (95% CI)^e^
**Age, y**						
18–40	26.2 (11)	2.39 (1.02–5.57)^f^	2.95 (0.73–11.94)	30.2 (13)	4.55 (1.89–10.97)^f^	4.37 (0.98–19.59)^f^
41–50	21.6 (11)	1.85 (0.81–4.24)	1.90 (0.48–7.53)	21.6 (11)	2.89 (1.18–7.05)^f^	1.10 (0.21–5.74)
51–60	35.0 (28)	3.62 (1.84–7.11)^f^	5.01 (1.66–15.13)^f^	23.8 (19)	3.27 (1.49–7.17)^f^	3.85 (1.11–13.34)^f^
61–70	29.2 (33)	2.77 (1.46–5.26)^f^	3.26 (1.18–8.98)^f^	17.5 (20)	2.23 (1.04–4.80)^f^	1.29 (0.38–4.43)
>70	12.9 (18)	1 [Reference]	1 [Reference]	8.7 (12)	1 [Reference]	1 [Reference]
**Sex**						
Female	22.4 (66)	0.78 (0.49–1.25)	0.63 (0.30–1.31)	17.6 (52)	1.02 (0.60–1.76)	0.71 (0.29–1.69)
Male	27.1 (36)	1 [Reference]	1 [Reference]	17.3 (23)	1 [Reference]	1 [Reference]
**Race**						
Nonwhite	38.1 (8)	2.06 (0.83–5.12)	1.37 (0.36–5.22)	19.0 (4)	1.15 (0.38–3.53)	1.33 (0.26–6.68)
White	23.0 (92)	1 [Reference]	1 [Reference]	17.0 (68)	1 [Reference]	1 [Reference]
**Ethnicity**						
Hispanic	30.0 (3)	1.38 (0.35–5.43)	0.00 (0.00–0.00)	30.0 (3)	2.07 (0.52–8.20)	0.99 (0.09–10.42)
Non-Hispanic	23.7 (98)	1 [Reference]	1 [Reference]	17.1 (71)	1 [Reference]	1 [Reference]
**Education**						
No college	19.8 (23)	0.72 (0.42–1.26)	0.73 (0.30–1.78)	14.8 (17)	0.78 (0.42–1.46)	0.82 (0.28–2.39)
Some college (1-3 y)	23.8 (25)	0.91 (0.53–1.58)	0.86 (0.37–1.99)	19.6 (21)	1.10 (0.61–2.00)	1.22 (0.47–3.15)
College graduate (≥4 y)	25.5 (52)	1 [Reference]	1 [Reference]	18.1 (37)	1 [Reference]	1 [Reference]
**Current smoker**						
Yes	20.0 (4)	0.80 (0.26–2.44)	0.48 (0.10–2.33)	15.0 (3)	0.80 (0.23–2.80)	0.14 (0.14–0.88)^f^
No	23.9 (95)	1 [Reference]	1 [Reference]	18.1 (72)	1 [Reference]	1 [Reference]
**Have hypertension**						
Yes	25.3 (61)	1.28 (0.81–2.02)	1.27 (0.57–2.83)	15.8 (38)	0.73 (0.45–1.21)	0.94 (0.37–2.40)
No	21.0 (39)	1 [Reference]	1 [Reference]	20.3 (38)	1 [Reference]	1 [Reference]
**Have diabetes**						
Yes	23.1 (21)	0.96 (0.55–1.66)	0.65 (0.27–1.53)	15.2 (14)	0.79 (0.42–1.49)	0.36 (0.12–1.07)
No	23.9 (80)	1 [Reference]	1 [Reference]	18.5 (62)	1 [Reference]	1 [Reference]
**Have high cholesterol**						
Yes	25.0 (56)	1.16 (0.74–1.81)	1.14 (0.56–2.32)	17.8 (40)	0.99 (0.60–1.63)	1.51 (0.66–3.49)
No	22.4 (45)	1 [Reference]	1 [Reference]	17.9 (36)	1 [Reference]	1 [Reference]
**History of heart attack or coronary artery disease**						
Yes	30.2 (16)	1.48 (0.78–2.78)	3.46 (1.19–10.06)^f^	25.9 (14)	1.77 (0.91–3.44)	2.92 (0.88–9.70)
No	22.7 (85)	1 [Reference]	1 [Reference]	16.5 (62)	1 [Reference]	1 [Reference]
**Body mass index^g^**						
>30.0	28.3 (40)	1.24 (0.70–2.19)	1.52 (0.64–3.62)	19.4 (28)	0.89 (0.47–1.68)	1.38 (0.51–3.76)
25.0–30.0	16.2 (28)	0.61 (0.32–1.16)	0.45 (0.18–1.10)	12.9 (18)	0.48 (0.23–1.01)^f^	0.51 (0.19–1.40)
18.0–24.9	24.2 (28)	1 [Reference]	1 [Reference]	20.9 (24)	1 [Reference]	1 [Reference]
**Self-reported health**						
Fair or poor	29.9 (26)	1.53 (0.90–2.59)	2.38 (0.78–7.25)	27.9 (24)	2.22 (1.27–3.89)^f^	2.60 (0.82–8.25)
Good or better	21.8 (73)	1 [Reference]	1 [Reference]	14.8 (50)	1 [Reference]	1 [Reference]
**Can go up and down stairs at a normal pace**						
Yes	24.1 (77)	1.07 (0.63–1.80)	2.34 (0.59–9.37)	17.1 (55)	0.88 (0.50–1.55)	1.39 (0.31–6.24)
No	22.9 (24)	1 [Reference]	1 [Reference]	19.0 (20)	1 [Reference]	1 [Reference]
**Can do chores**						
Yes	22.4 (80)	0.68 (0.38–1.21)	1.31 (0.38–4.50)	17.0 (61)	0.84 (0.43–1.63)	3.38 (0.82–14.04)
No	29.9 (20)	1 [Reference]	1 [Reference]	19.7 (13)	1 [Reference]	1 [Reference]
**Can walk ≥15 min**						
Yes	22.7 (77)	0.75 (0.44–1.28)	0.79 (0.22–2.78)	16.5 (56)	0.69 (0.38–1.23)	0.53 (0.13–2.23)
No	28.2 (24)	1 [Reference]	1 [Reference]	22.4 (19)	1 [Reference]	1 [Reference]
**Loneliness^h^**						
Yes	39.8 (53)	3.41 (2.14–5.46)^f^	5.66 (2.75–11.67)^f^	32.8 (44)	4.30 (2.54–7.28)^f^	4.81 (2.08–11.14)^f^
No	16.3 (46)	1 [Reference]	1 [Reference]	10.2 (29)	1 [Reference]	1 [Reference]
**Depression^i^**						
Yes	38.1 (8)	2.01 (0.81–5.00)	3.12 (0.52–18.77)	52.4 (11)	6.06 (2.47–14.88)^f^	4.67 (0.72–30.36)
No	23.4 (93)	1 [Reference]	1 [Reference]	15.4 (61)	1 [Reference]	1 [Reference]
**Anxiety^i^**						
Yes	32.5 (13)	1.61 (0.80–3.26)	0.26 (0.06–1.16)	41.5 (17)	3.99 (2.02–7.89)^f^	1.08 (0.27–4.27)
No	23.0 (87)	1 [Reference]	1 [Reference]	15.1 (57)	1 [Reference]	1 [Reference]

Abbreviations: CI, confidence interval; OR, odds ratio.

a Data obtained from a quality improvement survey administered to outpatients at a general internal medicine outpatient clinic at the Penn State Hershey Medical Center in September 2016.

b Described in survey as “A program where you and other people would play a game that can improve your problem-solving skills to help you feel less stressed.” Values in these columns include survey participants who expressed interest in the gameplay program only and in both programs.

c Described in survey as “A program where you and other people would discuss life problems with a mental health professional to help you feel less stressed.” Values in these columns include survey participants who expressed interest in the group interpersonal program only and in both programs.

d Denominators for percentages are all survey participants who answered questions for a given category. For example, 11 of 42 (26.2%) participants aged 18 to 40 were interested in using the gameplay program only or both programs.

e Based on multiple logistic regression analyses that assessed the independent contribution of each demographic or health-related explanatory variable, after adjusting for all other variables in the model.

f *P* < .05.

g Body mass index calculated as weight in kilograms divided by height in meters squared (kg/m^2^); weight and height were self-reported.

h Assessed by using a modified version of the UCLA Three-Item Loneliness Scale (7).

i Assessed by using the Patient Health Questionnaire-4 (6).

## Discussion

Consistent with our hypothesis, patients were significantly more interested in a gameplay-based problem-solving intervention than a group interpersonal therapy intervention. Traditional mental health care, such as group interpersonal therapy with a mental health professional described in the survey, may be perceived as having a greater overall burden (eg, less fun, requiring more resources, greater stigma) than a gameplay alternative. From a design thinking perspective — which encourages designers to consider the emotional experience of the end user (3) — distilling the core features of a highly effective treatment modality such as problem-solving therapy (4) into a more enjoyable social experience through gameplay, could offer promise for overcoming barriers to care.

A robust finding is that lonely patients were more interested in both mental health care programs than patients who were not lonely. From 2006 to 2016, the amount of time Americans spent socializing declined (8). In addition, the size of discussion networks (the set of people we turn to when we want to discuss important topics) and the average number of close friends appeared to have declined since 1985 (9). Because loneliness has implications for a wide range of physical and mental health outcomes (10), the development of social interventions, especially when they are fun and engaging with gameplay, can lead to the growth of new social relationships and their well-established benefits to health.

Although patients who screened positive for depression or anxiety were not significantly more interested in either program than patients who did not screen positive for these conditions, these patients did appear interested in these programs overall. For example, approximately one-third of patients with depression or anxiety were interested in the gameplay program. Gameplay appeared to have broader appeal to both groups (ie, those who had depression or anxiety and those who did not had similar levels of interest in gameplay) than traditional therapy. In contrast, traditional therapy appeared moderately more appealing to patients with depression or anxiety. This result was not unexpected, because the efficacy of traditional therapy is well established and patients with more severe symptoms may cautiously consider novel treatments. That we found greater interest in gameplay among patients with a history of heart attack or coronary artery disease than among patients without this history was unexpected; such a program may have been perceived as a safe (ie, nonstrenuous) and entertaining means of coping with stress, but data on such perceptions were beyond the scope of our study.

As young patients and lonely patients expressed greater interest in both programs than older and non-lonely patients, and patients with and without depression or anxiety expressed interest as well, a problem-solving gameplay program, given its broad appeal, may have utility as a stand-alone treatment or as a treatment used in conjunction with traditional approaches. Although a problem-solving gameplay program offers flexibility for tailoring activities to certain clinical populations, its application may be limited in other populations, such as people who have communication deficits or severe and persistent mental health symptoms (eg, schizophrenia spectrum, personality disorders). 

Our study has several limitations. Although many survey participants expressed interest in both programs, most expressed interest in neither. Because most reported relatively good health, healthy or nonstressed patients may have lacked sufficient justification for participation in the survey. The study sample consisted of fewer nonwhite participants and male participants than what is nationally representative, and it had relatively high rates of chronic conditions; thus, the generalizability of study findings to the US population may be limited. The potential for self-selection bias existed, as patients with severe symptoms may have opted out of the survey, and the method of survey administration may have unintentionally favored certain patient characteristics, such as motivation to volunteer. Finally, certain patient characteristics (eg, being younger or lonely) may have increased the susceptibility to demand characteristics (cues that create awareness among study participants about what researchers want to find and result in participants altering their responses to conform to researchers’ expectations); because of this potential bias, younger or lonely participants may have expressed interest in the therapies described when they actually had no interest.

This study highlights the opportunity for group mental health programs to be implemented across a diverse population of primary care patients. Although some primary care patients may lack interest in such programs for yet undetermined reasons, both programs offer potential to help patients cope with stress and improve mental health. Thus, a program based in gameplay, designed to be enjoyable, as preferred by patients in this study, should be developed and piloted in a randomized trial to gather feasibility and efficacy data.
